# CD151-α3β1 integrin complexes suppress ovarian tumor growth by repressing slug-mediated EMT and canonical Wnt signaling

**DOI:** 10.18632/oncotarget.2622

**Published:** 2014-10-22

**Authors:** Lauren A. Baldwin, John T. Hoff, Jason Lefringhouse, Michael Zhang, Changhe Jia, Zeyi Liu, Sonia Erfani, Hongyan Jin, Mei Xu, Qing-Bai She, John R. van Nagell, Chi Wang, Li Chen, Rina Plattner, David M. Kaetzel, Jia Luo, Michael Lu, Dava West, Chunming Liu, Fred R. Ueland, Ronny Drapkin, Binhua P. Zhou, Xiuwei H. Yang

**Affiliations:** ^1^ Department of Pharmacology and Nutritional Science, Department of Molecular and Cellular Biochemistry, and Markey Cancer Center, University of Kentucky, Lexington, KY; ^2^ Division of Gynecologic Oncology, Department of Obstetrics and Gynecology, Department of Pathology & Laboratory Medicine, University of Kentucky, College of Medicine, and Markey Cancer Center, University of Kentucky, Lexington, KY; ^3^ Department of Biochemistry and Molecular Biology, University of Maryland School of Medicine, Baltimore, Maryland; ^4^ Department of Biomedical Science, Florida Atlantic University, Boca Raton, Florida, United States of America; ^5^ Department of Cancer Biology and Pathology, Dana-Farber Cancer Institute and Harvard Medical School, Boston, MA

**Keywords:** CD151, integrin, EMT, tumor growth, ovarian cancer

## Abstract

Human ovarian cancer is diagnosed in the late, metastatic stages but the underlying mechanisms remain poorly understood. We report a surprising functional link between CD151-α3β1 integrin complexes and the malignancy of serous-type ovarian cancer. Analyses of clinical specimens indicate that CD151 expression is significantly reduced or diminished in 90% of metastatic lesions, while it remains detectable in 58% of primary tumors. These observations suggest a putative tumor-suppressing role of CD151 in ovarian cancer. Indeed, our analyses show that knocking down CD151 or α3 integrin enhances tumor cell proliferation, growth and ascites production in nude mice. These changes are accompanied by impaired cell-cell contacts and aberrant expression of E-cadherin, Mucin 5AC and fibronectin, largely reminiscent of an epithelial to mesenchymal transition (EMT)-like change. Importantly, Slug, a master regulator of EMT, is markedly elevated. Knocking down Slug partially restores CD151-α3β1 integrin complex-dependent suppression of cell proliferation. Moreover, disruption of these adhesion protein complexes is accompanied by a concomitant activation of canonical Wnt signaling, including elevated levels of β-catenin and Axin-2 as well as resistance to the inhibition in β-catenin-dependent transcriptional complexes. Together, our study demonstrates that CD151-α3β1 integrin complexes regulate ovarian tumor growth by repressing Slug-mediated EMT and Wnt signaling.

## INTRODUCTION

Ovarian cancer is the leading cause of death among gynecologic cancers and the fifth most common cause of cancer related death in the United States. Nearly 70% of newly diagnosed ovarian tumors are in the late, metastatic stages of disease [1]. Clinically, treatment for serous (the most common cell type) ovarian cancer is surgical resection followed by combination platinum- and taxol-based chemotherapies. Unfortunately, 20% of patients are resistant to this regimen and most patients that are initially responsive will become resistant and ultimately succumb to their disease [2, 3]. Despite the aggressive yet predictable route of tumor dissemination and high failure rate of current therapies, the molecular and cellular mechanisms underlying this cancer type remain poorly defined [4, 5]. Hence, a better mechanistic understanding of ovarian cancer progression and metastasis is urgently needed in order to develop more effective target-based therapies.

As a family of heterodimeric adhesion receptors, integrins are widely recognized for their role in controlling tumor cell interactions with the extracellular matrix (ECM) and neighboring cells in human epithelia-origin ovarian cancer [1, 6]. Notably, elevated expression of RGD-based integrins, including αvβ3 and α5β1, along with their ligands vitronectin and fibronectin, are strongly associated with accelerated ovarian tumor cell proliferation and progression [7, 8]. Such functional links appear to be related to a concomitant disruption in E-cadherin-mediated cell-cell contacts and the induction of epithelial to mesenchymal transition (EMT) [9, 10]. For instance, in SK-OV-3ip cells, the expression of fibronectin and its receptor α5β1 integrin are markedly elevated upon removal of E-cadherin [7]. Thus, there is strong evidence that integrins contribute to the development and progression of human ovarian cancer by impacting cell-ECM and E-cadherin-dependent cell-cell interactions.

In contrast to RGD-based integrins, the role of laminin-binding (LB) integrins, including α3β1, α6β1 and α6β4, is less defined, particularly with regards to the progression of human ovarian cancer. In some studies α6β4 integrin is suggested to be a promoter of ovarian cancer cell invasiveness [11]. In contrast, α3β1 integrin appears to contribute to the maintenance of stable cell-cell contact by regulating the activity of small GTPase Rho A, thereby conferring a potential tumor-suppressive function [12, 13]. In fact, several studies have reported that expression of α3 integrin is inversely correlated with the metastatic potential of human ovarian cancer, particularly regarding peritoneal dissemination [14, 15]. Thus, LB integrins are likely a central player in controlling the malignancy of ovarian cancer.

Recently, CD151, a master regulator of LB integrin function and signaling, has also been implicated in the progression of ovarian cancer. As a member of the tetraspanin family with four transmembrane domains, CD151 plays a variety of roles in multiple stages of human cancer development and progression [12, 16, 17]. For instance, CD151 has been suggested to promote tumor cell invasiveness and drug sensitivity through regulation of α6 integrins (α6β1 and α6β4) [18, 19]. A similar role has been recently implicated for CD151 in the progression of ovarian cancer [20]. However, there is also evidence that attenuation of CD151 expression impairs α3β1 integrin-dependent cell adhesion and motility over laminin substrates in a number of human carcinoma types. These effects are frequently accompanied by a concomitant disruption in E-cadherin-mediated cell–cell contacts [12, 17], implying a putative tumor-suppressive role for CD151-α3β1 integrin complexes. Clinically, the direct sloughing to the peritoneal cavity is considered a primary disseminating route for ovarian cancer, as opposed to vascular or lymphatic trafficking in other carcinoma types, such as breast or prostate cancer [1]. In this regard, adhesion complexes, such as CD151-α3β1 integrin, may have a strong impact on ovarian cancer progression and metastasis through controlling cell-cell contact. Thus, available observations on the function of CD151 and its associated LB integrins in human ovarian cancer are conflicting and need clarification.

Here we report a functional role of CD151 and its associated α3β1 integrin in human serous-type ovarian cancer. Human tumor tissue arrays (TMA) were adopted for evaluation of the clinical relationship between CD151 and ovarian tumor malignancy. Xenograft and signaling analyses were then conducted to delineate the functional cascade of CD151-α3β1 integrin complexes during ovarian tumor growth and progression. Our results indicate that CD151-α3β1 integrin complexes suppress ovarian cancer malignancy largely by counteracting EMT-like processes as well as inhibiting Slug activation and canonical Wnt signaling. Hence, our study for the first time illustrates that CD151-α3β1 integrin complexes and associated pathways regulate ovarian tumor growth and progression and may serve as potential therapeutic targets against this lethal human cancer.

## RESULTS

### CD151 expression is inversely associated with the malignancy of human serous-type ovarian cancer

**Figure 1:**
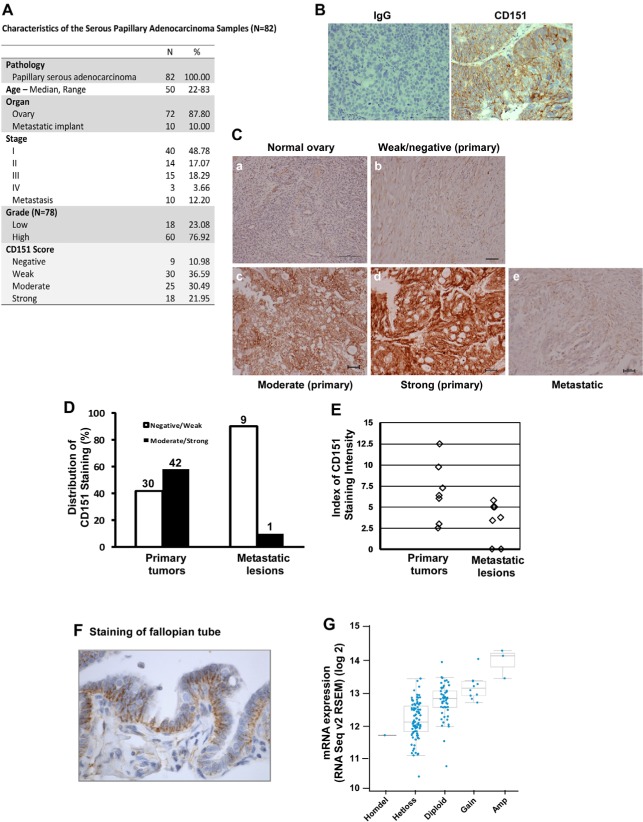
CD151 expression in human serous-type ovarian tumors A Clinical parameters of TMA harboring primary tumors and metastatic lesions across age groups and grades. B Validation of IHC staining of CD151 in human ovarian tumors. *Left panel*: Non-immune IgG control; *Right panel*: CD151antibody. C Representative images of CD151 staining of varying intensity. *a*, normal ovary. *b*-*d*: ovarian tumors. *e*: metastatic lesion. Scale bar: 100 μm. D Distribution of CD151 staining in primary tumors and metastatic lesions (Biomax, n = 82). Based on the antibody staining of tissue sections, tumor samples were divided into the negative/weak or moderate/strong group. The numbers of tumor samples per group were indicated. E Analyses of CD151 expression with in-house TMA housing 7 pairs of matched human primary and metastatic ovarian tumors. Patient characteristics described in prior studies [64]. F CD151staining in fallopian tube. G Plot of gene copy number vs mRNA expression of CD151 in the TCGA ovarian cancer samples was generated as previously described [22]. *Homdel*: Homozygous deletion; *Hetloss*: Heterozygous loss; *Gain*: Copy number increase; *Amp*: Amplification.

To evaluate the clinical significance of CD151 expression in human ovarian cancer, IHC analyses were conducted on a tumor tissue array (TMA) harboring normal ovarian tissue, as well as papillary serous adenocarcinomas varying in grade and metastatic status (Fig. 1A). By staining with a CD151-specific monoclonal antibody (Fig. 1B), we found that CD151 was only weakly expressed in the epithelium of the normal human ovary (Fig. 1C). In contrast, significant CD151 staining was detected on the plasma membrane of tumor cells, and displayed a wide range of intensities between tumor tissue samples (Fig. 1C). Notably, a moderate or strong degree of expression of CD151 was detected in 58% of primary ovarian tumors, but in < 10% of metastatic tumors (p < 0.0055) (Fig. 1D). A similar trend was found in another independent TMA for which matched primary and metastatic tumor samples from 7 individual patients were evaluated (Fig. 1E) ( p < 0.024). It is also worth noting that the fallopian tube, another site for serous –type ovarian cancer origin [21], displayed a basal-lateral staining pattern for CD151 (Fig. 1F). Moreover, consistent with decreased CD151 protein in metastatic tumors was evidence of the heterozygous loss and a marked reduction in mRNA for this gene in the majority of TCGA ovarian tumor samples (Fig. 1G) [22]. There was, however, minimal association between CD151 expression and ovarian tumor grade or stage, which contrasts to prior reports on the expression of this protein in other cancer types [19, 23, 24]. Together, these observations indicate a significant inverse association between CD151 expression and metastatic status, suggesting a potential suppressive role of CD151 during ovarian tumor progression.

### CD151 suppresses ovarian cancer cell proliferation, tumor growth, and ascites production

**Figure 2:**
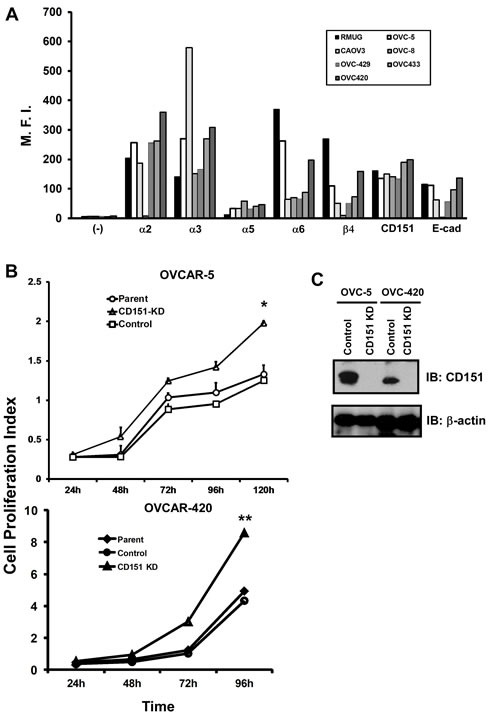
CD151 expression and its role in ovarian tumor cell proliferation A The surface expression of CD151 and its molecular partners in cultured human ovarian cancer cell lines. The mean fluoresce intensity (M.F.I) represents an average of fluorescence intensity measurement from a total of 10,000 cells on flow cytometry. Semi-confluent human ovarian cancer cell lines, including OVCAR-5, OVCAR-8, OVCAR-420, OVCAR-429, OVCAR-433 and CAROV3 were detached by non-enzymatic buffer and incubated with anti-CD151 (5C11), α3 (X8), α6 (GoH3), β4 (3E1), α2 (IIE10) and α5 (BIIG2). B Changes in cell proliferation over indicated periods of time were determined using the MTT assay. Cell proliferation index was calculated on the basis of OD reading. *: p < 0.05; **: p <0.01. C CD151 knockdown in human ovarian cancer cell lines, OVCAR-5 and OVCAR-420. Monoclonal antibody 1A5 was used for CD151 blotting. β-actin was blotted as loading control.

Next, we investigated the functional role of CD151 using cultured human serous-type ovarian tumor cell lines. Initially, our FACS analyses indicated that the degree of surface expression of CD151 and its associated LB integrins (α3, α6 and β4) were highly variable between ovarian cancer cell lines, similar to their RGD-based counterparts (e.g., α2β1 or α5β1 integrin) (Figure 2A). Interestingly, most of these lines, except OVCAR-8, displayed typical epithelia-like morphologies as reflected by a high expression of cell surface E-cadherin.

**Figure 3:**
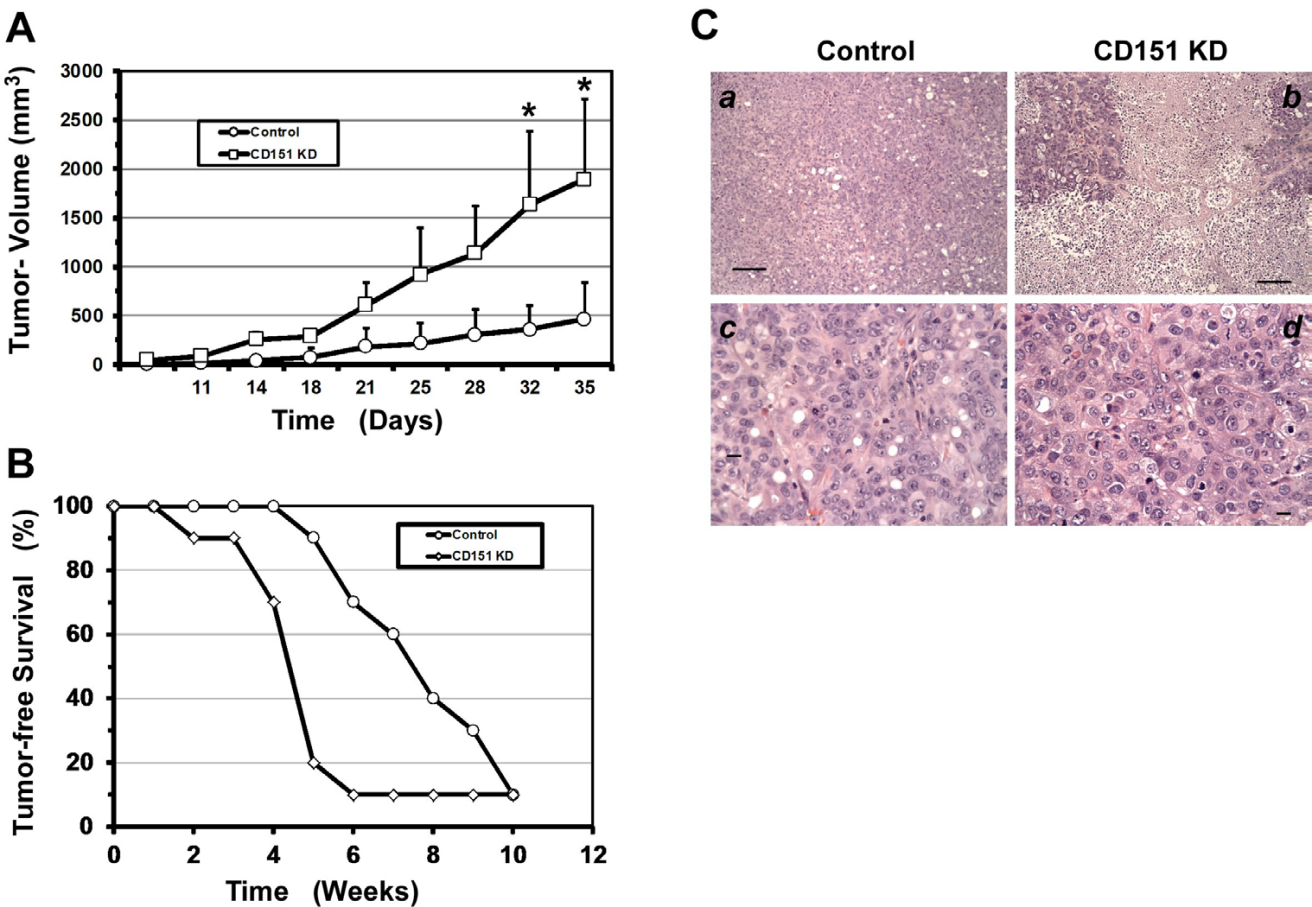
CD151 ablation enhances ovarian tumor growth and ascites production A Ectopic analyses of ovarian tumor growth. The back flanks of nude mice were subcutaneously injected with control and CD151-ablated OVCAR-5 cells respectively and subsequently measured for tumor appearance and growth over the indicated periods of (n =10). B Kaplan-Meier survival curve. Equal numbers of immuno-deficient mice were given an intra-peritoneal injection of OVCAR-5 cells with or without CD151 ablation (n = 10). C H & E stained sections of ovarian tumors derived from subcutaneously injected OVCAR-5 cells. a, c: control b, d: CD151 knockdown. Scale bar: 50 μm.

Because OVCAR-5 and OVCAR-420 displayed significantly higher expression of CD151 and LB integrins (Figure 2A), these lines were selected as our representative models for subsequent functional analyses. As shown in Fig. 2A, upon CD151 ablation by stable expression of a well-defined shRNA from prior studies [18, 19, 25, 26], the proliferation of OVCAR-5 and OVCAR-420 cells increased by 60% and 53%, respectively. However, there were minimal changes in cell motility or apoptosis of tumor cells upon CD151 removal (data not shown). Given the strong influence of CD151 ablation on cell proliferation (Fig. 2B), we next evaluated the role of CD151 in ovarian tumor growth and metastasis by applying *ex vivo* xenograft models in immunocompromised mice. As shown in Fig. 3A, tumors derived from subcutaneously injected CD151-deficient OVCAR-5 cells grew significantly faster in mice, compared to the control group (p < 0.05). Furthermore, there was enhanced ascites production and a concomitant decrease in tumor-free survival in mice injected with CD151-deficient tumor cells (4.3 vs 7.5 weeks, p < 0.005) (Fig. 3B). Moreover, the tumors derived from CD151-ablated OVCAR-5 cells displayed typical high-grade serous tumor histomorphologic features. Notably, these ovarian tumors had apparent nuclear pleiomorphism, prominent nucleoli, a high mitotic index, and extensive necrosis (up to 50% of tumor volume). Collectively, data from our *in vitro* and xenograft analyses consistently suggest a strong suppressive role of CD151 in ovarian tumor growth and progression.

### Impact of CD151 removal on cell-cell contact and α3β1 integrin-associated protein complexes

**Figure 4:**
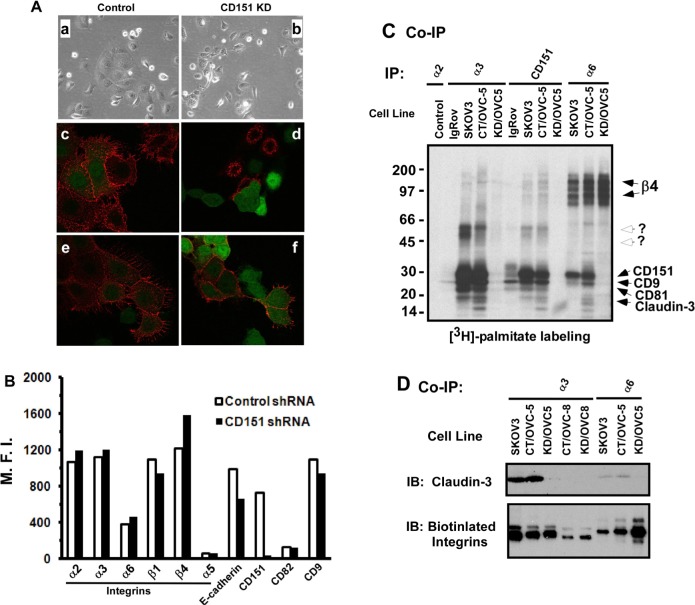
Impact of CD151 ablation on ovarian tumor cell-cell contact A Typical morphologies and antibody staining of OVCAR-5 cells with or without CD151 knockdown (control or KD). a-b: Image of cells cultured for 17-24 hours; c-d staining of cell surface CD151; e-f staining of cell surface E-cadherin. c-f, cells were stained alive on ice with antibodies prior to being fixed. *a, c, e*: Control; *b, d, f*: CD151 knockdown (KD). *Red*- antibody staining; *Green*: GFP. B Change in expression of surface molecules in OVCAR-5 cells upon CD151 ablation. M.F.I ( Mean fluorescence intensity) was determined by FACS analyses. C Radiography of [^3^H]-labeled tetraspanin- and integrin-associated protein complexes. Reciprocal co-immunoprecipitation (Co-IP) of CD151 and LB integrins was conducted for ovarian cancer cell lines IgRov, SKOV3 and OVCAR-5. Tumor cells expressing control (CT) or CD151 shRNA (KD) were included. Antibodies used for Co-IP were IIE10, X8, GÖH3 and 5C11 for integrin α2, α3, α6 and CD151, respectively. D Analysis of the physical interaction between LB integrins and claudin-3. Multiple ovarian tumor cell lines (SKOV3, OVC-5 and OVCAR-8) were labeled with biotin, followed by lysing in 1.0% Brij96 buffer as previously described [65]. After Co-IP with either α3 or α6 antibodies (X8 or GÖH3), the immune complexes were blotted with claudin-3-specific antibody or Avidin-conjugated HRP.

There is increasing evidence that the accelerated growth and progression of human ovarian carcinoma is associated with diminished or reduced cell-cell contact [1, 21]. CD151 and its associated α3β1 integrin are pivotal players in stabilizing cell-cell contacts in normal and malignant epithelial cells [12, 17]. We therefore tested the notion that CD151 and α3β1 integrin act together to suppress ovarian tumor growth and progression by influencing tumor cell-cell contacts within the tetraspanin-enriched membrane microdomain (TEMM). As shown in Fig. 4A, CD151 removal in OVCAR-5 cells led to a switch from the epithelia-like sheet to a scattered fibroblast-like appearance, accompanied by a marked reduction in surface expression of E-cadherin, compared to the control cells. Similar changes were also detected in several other ovarian tumor cell lines, including OVCAR-420 and CAROV3 (data not shown). In line with these observations is a marked decrease in the surface expression of E-cadherin when CD151 was knocked down by 90%, according to our FACS analyses (Fig. 4B). On average, the surface level of E-cadherin in CD151-knockdown cells decreased by 33.5% ± 1.0% (n = 3, p < 0.001). These results are consistent with prior reports on the effect of CD151 ablation in other types of human carcinoma cells [12, 17]. Because E-cadherin-associated cell-cell contacts confer an inhibitory role in ovarian tumor cell growth and dissemination [27], our data imply that CD151 may suppress ovarian cancer malignancy by stabilizing cell-cell contacts between carcinoma cells.

**Figure 5:**
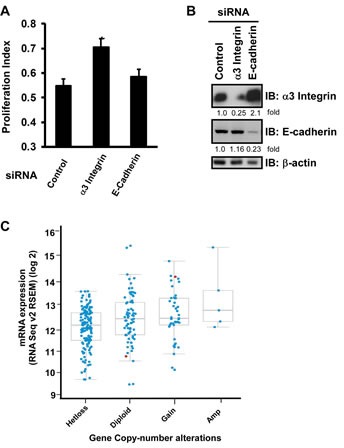
CD151-associated α3 integrin suppresses ovarian tumor cell proliferation A Cell proliferation Index (mean + SEM, n =5). Cells seeded into 24 well plates were treated with indicated siRNA and 48 hrs later analyzed by MTT assay. B Immunoblotting of α3 integrin and E-cadherin protein in OVCAR-5 cells after being treated siRNA for 72 hrs. The fold change in protein level was calculated on the bases of densitometry analyses. C Plot of gene copy number vs mRNA expression of α3 integrin in TCGA ovarian cancer samples was generated as previously described [22]. *Hetloss*: Heterozygous loss; *Gain*: Increase in copy number; *Amp*: Amplification.

Since tetraspanin molecules like CD151 are known to function within TEMM [28, 29], we next evaluated the impact of disrupting CD151 on the integrity of LB integrin-enriched protein complexes in ovarian cancer cells. Our co-immunoprecipitation analyses with [^3^H]-palmitate-labeled tumor cells indicate that a number of proteins, including CD9 and CD81, were no longer associated with α3β1 integrin in the absence of CD151 (Fig. 4C). Interestingly, we also detected a marked decrease in the association with claudin-3 (Fig. 4D), a tight junction protein and a driver of ovarian tumor growth and metastasis [30, 31]. In line with these observations is that knockdown of α3β1 integrin enhanced tumor cell proliferation (Fig. 5A). However, removal of E-cadherin, had little effect on the proliferation of OVCAR-5 cells, and was accompanied by a concomitant increase in α3 integrin expression (Fig. 5B). In line with a suppressive role of α3 integrin is a trend towards reduced mRNA expression of this gene in ovarian tumors (Fig. 5C), similar to the prior analysis of CD151 (Fig. 1E). Collectively, these results provide evidence that CD151 and α3β1 integrin act together to suppress human ovarian tumor growth and dissemination by regulating the integrity of cell-cell contacts among tumor cells.

### CD151 removal enhances the activation of EMT-inducing transcription factor Slug

**Figure 6:**
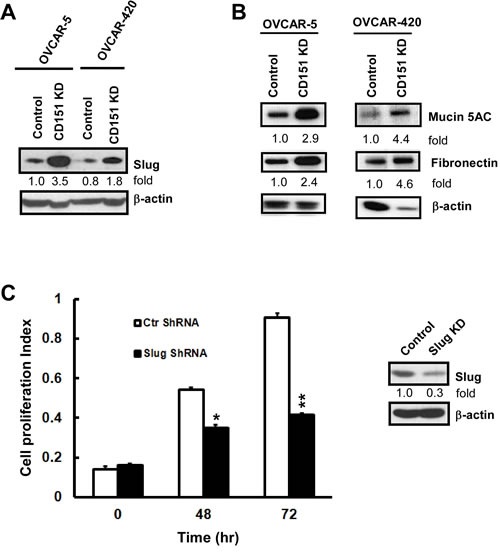
A functional link between CD151, EMT and Slug in ovarian cancer cells A Effect of CD151 ablation on Slug expression in OVCAR-5 and OVCAR-420 cells. Cells were lysed in RIPA buffer and blotted with indicated antibodies. B Induction of Fibronectin and Mucin5AC in CD151-deficient tumor cells. OVCAR-5 and OVCAR-420 cells with or without CD151 knockdown (KD) were lysed in RIPA buffer and blotted with indicate antibodies. C Effect of Slug knockdown on the proliferation of CD151-deficient OVCAR-5 cells (*left panel*). Blotting of Slug protein in control and knockdown cells (*right panel*). Bottom panel, proliferation index of control and Slug-knockdown cells was assessed by the MTT assay. For A-C, the fold change in protein level was estimated according to densitometry analyses.

Building on our previous finding where CD151 removal gave rise to an EMT-like morphology in ovarian cancer cells (Fig. 4), we evaluated the state of EMT-inducing factors. While there was minimal change in the expression of Twist, Snail or ZEB protein upon CD151 ablation (data not shown), expression of Slug proteins increased by 1.8-3.5-fold in OVCAR-5 and OVCAR-420 tumor cells (Fig. 6A). Furthermore, our genome-wide expression profiling analyses indicated that CD151 removal led to a minimal change in the mRNA of Slug (data not shown). Hence, we speculate that the increased Slug expression in CD151-deficient ovarian tumor cells may be largely attributed to post-translational regulation. In supported of elevated level of Slug protein was the change in a number of EMT-associated genes in CD151-deficient OVCAR-5 cells, including fibronectin (FN1) and Mucin 5Ac (MUC5AC) [8, 32] (Table 1). Our subsequent analyses indicated that increased expression of Mucin 5Ac and fibronectin also occurred at the protein level (Fig. 6B). Since Slug is strongly implicated in regulating epithelial cell proliferation [33], we next tested if CD151 removal impacted ovarian tumor cell properties in a Slug-dependent manner. As shown in Fig. 6C, Slug knockdown indeed led to a marked decrease in the proliferation of CD151-deficient OVCAR-5 cells. Taken together, these observations consistently suggest that CD151 plays a suppressive role in ovarian carcinoma growth largely by counteracting Slug-mediated EMT-like process. This includes alterations in cell-cell contact and a phenotypic switch to the mesenchymal phenotype as reflected by cell scattering and increased expression of fibronectin and Muc5Ac.

**Table 1:** List of differentially expressed genes in OVCAR-5 ovarian cancer cells upon CD151 removal Fold changes were obtained by calculating the ratio of the mean of expression intensity for each gene. P values on the fold change were determined using paired t-test.

Gene	Accession	Function	Ratio of Sh CD151/control	P-value
MUC5AC	AW192795	proliferation	34.99	0.002573
KIAA1199	AB033025	cellular proliferation	17.57	0.031699
RNF43	NM_017763	promotes cell growth	6.92	0.018189
S100A2	NM_005978	cell cycle progression and differentiation	5.25	0.028039
LGR4	NM_018490	Epithelial cell proliferation	3.79	0.006977
ID-1	D13889	tumour growth and angiogenesis	3.54	0.108128
LIF	NM_002309	cell growth by inhibiting differentiation	2.83	0.017261
KYNU	D55639	catabolism of Trp metabolism	5.4	0.030945
SLC39A4	NM_017767	zinc metabolism	5.23	0.010953
CD55	CA448665	protein metabolism	3.78	0.030651
AKR1B10	NM_020299	metabolism	3.38	0.049357
ALDH3B1	BC002553	aldehydes metabolism	3.34	0.018712
GCNT3	NM_004751	mucin-type biosynthesis	3.34	0.04714
SLC16A3	AL513917	Lactic acid and pyruvate transport	3.33	0.072933
CAMK2N1	NM_018584	Ca^++^ metabolism	2.93	0.022496
SAT1	M55580	Spermidine metabolism	2.83	0.024851
Sox4	AL136179	Cell fate	4.9	0.024132
FOXP1	AW080845	cell fate	4.24	0.01093
FN1	BC005858	cell motility	4.03	0.039951
JAG1	U73936	cell fate	2.69	0.036003
SLC1A1	AW235061	transporting glutamate	4.13	0.030336
RGS2	NM_002923	Inhibits signal transduction	3.42	0.017092
ANP32A	NM_006305	transcriptional regulation	−2	0.472994
ANKRD12	X80821	transcriptional regulation	−2.19	0.027674
SNF1	NM_014840	Survival, invasion and metastasis	−2.34	0.038872
SGEF	AI989530	protein binding	−2.45	0.013114
Claudin-11	AW264204	Tight junciton	−2.8	0.034391
BMP2K	AI735391	differentiation.	−3.28	0.02009
COTL1	AJ227860	regulate the actin cytoskeleton.	−3.37	0.007368
RBMS1	AA428240	protein binding	−3.39	0.062653
ST6GALNAC1	NM_030965	glycosylaiton	−4.98	0.027494
NMRAL1	AI080701	Redox sensor protein.	−5.19	0.011539
CD151	NM_004357	cell adhesion	−7.21	0.179191

### CD151 removal in ovarian cancer cells is accompanied by an induction of Wnt signaling

**Figure 7:**
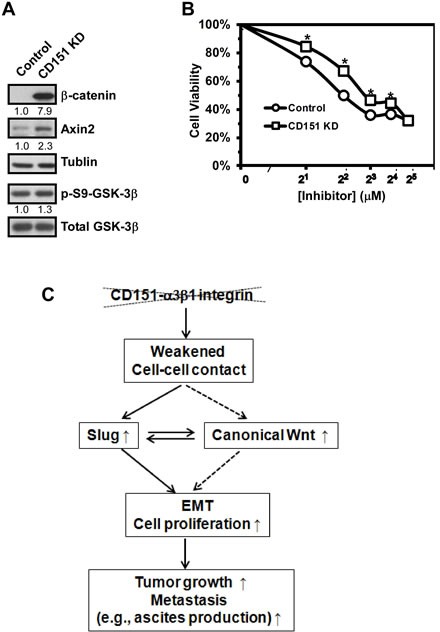
Induction of canonical Wnt signaling in ovarian cancer cells upon CD151 removal A Blotting of key signaling intermediates in Wnt pathways in OVCAR-5 cells expressing control and CD151-specific shRNA. Tumor cells were lysed in RIPA buffer and blotted with antibodies against Wnt signaling molecules (i.e., β-catenin and Axin-2) as well as total and phosphorylated forms of GSK-3β kinase. Tublin was blotted as control. The fold change in protein level was estimated according to densitometry analyses. B Impact of CD151 knockdown on tumor cell viability in response to the treatment of ICG-001, an inhibitor disrupting the transcription within the canonical Wnt/β-catenin/TCF/CBP signaling axis. OVCAR-5 cells with or without CD151 knockdown were seeded into 48 well plates and then treated with varying dosages of Wnt inhibitor for 72 hours prior to being analyzed by MTT assay. C Schematic illustration of functional roles of CD151-α3β1 integrin complexes in ovarian cancer.

To understand how CD151 regulated Slug in ovarian cancer cells, we turned our attention to Wnt signaling, as it has been strongly connected to the activation of Slug in human carcinomas [34, 35]. In fact, our DNA array analyses revealed marked changes in a number of Wnt pathway-related genes. Notably, upon CD151 ablation, KIAA1199 and RNF43, two newly identified regulators of Wnt signaling [36-38], increased by 7- and 18-fold at the mRNA level, respectively (Table 1). Also, similar changes in the RNF43 gene occurred at the protein level (data not shown). Moreover, CD151-deficient ovarian cancer cells displayed a 2-8-fold increase in β-catenin and Axin-2 proteins, two key signaling intermediates downstream of the canonical Wnt pathway (Fig. 7A). Our luciferase reporter assay with FOPFLASH reporter construct, however, revealed little change in the transcriptional activity of β-catenin/TCF complexes upon CD151 removal, regardless of Wnt3A stimulation (data not shown), implying a constitutive activation of β-catenin-mediated signaling. In line with these observations is that upon treatment with ICG-001, an inhibitor specifically disrupting the transcription mediated by β-catenin/CBP complexes [39], CD151-deficient OVCAR-5 cells displayed significantly higher viability over a range of 4 to 126 μM, compared to the control (Fig. 7B). It is also worth noting that there was minimal change in the activation in PI3K/AKT- or RAS/MAPPK-mediated pathways in ovarian carcinoma cells upon CD151 ablation (data not shown). Collectively, these data indicate a novel signaling cross-talk between CD151 and the canonical Wnt/β-catenin/CBP axis-mediated pathway in ovarian cancer cells.

## DISCUSSION

This study presents first clinical and experimental evidence of the suppressive role of CD151 and its associated α3β1 integrin in serous-type ovarian cancer. IHC and genomic analyses of human ovarian tumor tissues suggest an inverse correlation between CD151 expression and tumor metastasis. Consistent with this observation is that CD151 ablation markedly impacts ovarian tumor cell proliferation as well as tumor growth and ascites production in immuno-compromised mice. Furthermore, E-cadherin expression and cell-cell contacts in ovarian tumor cells were markedly altered upon disruption of CD151 and/or α3β1 integrin. In line with this EMT-like alteration is the concomitant activation of transcription factor Slug and canonical Wnt signaling. Taken together, our results demonstrate that CD151 and α3β1 integrin act together to suppress ovarian tumor cell growth and malignancy largely by controlling cell-cell contacts, Wnt signaling and Slug activation (Fig. 7C).

### CD151- α3β1 integrin complexes suppress tumorigenesis by counteracting the EMT-like process

Our analyses of clinical specimens, tumor cell activities and xenograft models support a tumor-suppressive role of CD151-α3β1 integrin complexes in ovarian cancer. This finding is consistent with the recent studies on CD151 in human endometrial and colon cancers [40, 41]. In addition, a similar suppressive role in tumor progression has been described for α3β1 integrin [42, 43]. These observations differ from the reports on the function of these molecules in other cancer types, including breast and prostate cancer [19, 44, 45]. It has been suggested that the complex roles of LB integrins during carcinogenesis are dependent on the oncogenic contexts [29, 46]. In this regard, the tumor-suppressive function of CD151-integrin complexes described in the current study may be partially associated with unique oncogenic activations in human serous ovarian cancer revealed by recent genome-wide analyses [4, 47].

Aside from genetic factors, the tumor-suppressive role of CD151-α3β1 integrin complexes in ovarian cancer may be related to their roles in maintaining stable cell-cell contact [12, 17]. Our data show that intact CD151-α3β1 integrin complexes inhibit the induction of the EMT-like process (Figs. 4-6), a critical driver of ovarian cancer progression and metastasis [1, 9]. Interestingly, in addition to decreased expression of E-cadherin, CD151 removal leads to a marked induction in fibronectin (FN) (Figs.4-6), a strong promoter of ovarian cancer malignancy [7, 8, 48]. Such functional shift from E-cadherin- and LB integrin-mediated cell-cell contact to α5β1 integrin-mediated cell-ECM (i.e., fibronectin) interaction may also explain why there is elevated activation of small GTPase RhoA in CD151-deficient carcinoma cells recently described by Johnson et al [12, 29]. In support of this notion is that the engagement of fibronectin with its primary receptor, α5β1 integrin, is more effective in activating RhoA, compared to the interaction between laminins and their integrin receptors [49]. Consequently, our study raises the possibility that CD151-α3β1 integrin complexes impact ovarian cancer malignancy largely by repressing the EMT-like process and potentially the RhoA activation from fibronectin-α5β1 integrin-mediated signaling.

Data from the current study also suggest that the induction of the EMT-like event upon the disruption of CD151-integrin complexes may result from the activation of transcription factor Slug as well as increased cytoplasmic and nuclear expression of β-catenin. In fact, as master regulators of EMT, increased transcriptional activities of Slug and β-catenin have been associated with a broad spectrum of tumorigenic processes [50, 51]. Consistent with our observations (Figs. 3-5) is the promoting role of these two transcription factors in ovarian cancer development and malignancy described by multiple studies [9, 52-54]. At the present time, however, how CD151 removal leads to the increased expression or activation of Slug and β-catenin in ovarian cancer remains unclear.

### Functional links between CD151- α3β1 integrin complexes and oncogenic pathways

One of the surprising observations from the current study is the inverse functional link between CD151-α3β1 integrin complexes and the signaling of canonical Wnt pathways in ovarian cancer. This finding is consistent with recent studies on tetraspanin molecules, such as tetraspanin CD9, which suppresses Wnt signaling-dependent tumor cell proliferation and growth [55, 56]. In addition, these studies all point to the repression of β-catenin-dependent canonical Wnt signaling by tetraspanin molecules. Mechanistically, since minimal change was detected in the mRNA levels of Frizzed or LRP5/LRP6 in our study, the regulation of canonical Wnt signaling by CD151-integrin complexes may not occur at the receptor level. This observation is also in contrast to a recent report on the increase in LRP5 mRNA in CD151-null mouse tissues [57], raising the possibility of a tissue-specific effect of CD151 on Wnt signaling. Intriguingly, we also detected a marked elevation in the expression of KIAA1199 and RNF43 in CD151-deficient ovarian tumor cells (Table 1), two newly identified negative regulators of Wnt signaling [38, 58], implying a potential feedback mechanism related to CD151 function. Collectively, the marked changes in these important effectors and regulators of Wnt signaling underscore the complex signaling roles of CD151-α3β1 integrin complexes in ovarian tumor growth and progression.

### Sequestering of pro-tumorigenic regulators within TEMM by CD151-integrin complexes

Additional evidence supporting the suppressive role of CD151-α3β1 integrin complexes in ovarian cancer comes from the loss of tumor-promoting molecules within CD151-deficient TEMM, such as CD9 and claudin-3 (Fig. 4). In fact, both CD9 and claudin-3 proteins are present in TEMM on tumor cells [28, 29, 59] and have been strongly linked to cancer malignancy [30, 31, 60]. In particular, CD9 has been shown to promote ovarian cancer cell dissemination by influencing integrin activation [31]. It has also been suggested that claudin-3 drives ovarian tumor progression and metastasis by controlling cell-cell contacts and Wnt signaling [61]. Conceivably, CD151 may regulate ovarian tumor growth and dissemination, at least in part, by sequestering the function of tumor-promoting molecules such as CD9 and claudin-3 within the TEMM in ovarian tumor cells.

### Clinical significance of CD151-integrin complexes as ovarian tumor suppressors

Results from the current study have important implications for the diagnosis and treatment of human ovarian cancer. In particular, we found that two secreted proteins, Mucin 5Ac and fibronectin, exhibit elevated expression upon the disruption of CD151-α3β1 integrin complexes (Table 1 & Fig. 6). In addition, like CA125, a common biomarker for ovarian cancer [62, 63], Mucin 5Ac belongs to the Mucin family, and has been implicated in promoting ovarian cancer progression [32]. Hence, evaluating these CD151-α3β1 integrin complex-associated effectors as new biomarkers may aid in the future detection or diagnosis of human ovarian cancer malignancy. Moreover, the pro-malignancy factors or pathways counteracted by CD151 and α3β1 integrin may serve as therapeutic targets against ovarian cancer. Based on our observations (Figs. 6-7), the inhibitors of the transcriptional factor Slug or β-catenin-dependent canonical Wnt signaling pathway (Figs. 6-7) are potentially effective in disrupting the malignancy of ovarian cancer.

In summary, our study demonstrates a suppressive role of CD151 and associated α3β1 integrin in ovarian tumor cell proliferation, growth and ascites production. Mechanistically, CD151 conveys its tumor-suppressing function largely by stabilizing α3β1 integrin- and E-cadherin-mediated cell-cell contact, while counteracting the EMT-like process through repressing the activation of Slug and canonical Wnt signaling. As such, our study has identified a new set of molecular pathways and regulators as candidate biomarkers and therapeutic targets for the treatment of aggressive serous-type ovarian cancer.

## METHODS

### Human ovarian TMA, cell lines and culture

TMA harboring paraffin-embedded human ovarian tumors prepared from a broad spectrum of ovarian cancer patients (Supplementary Table S1) were obtained from Biomax (NY). The in-house TMA containing matched primary tumors and metastatic lesions from individual patients were described in a prior study [64]. Human ovarian cancer cell lines, including CAOV3, OVCAR-5, OVCAR-8, OVCAR-420, OVCAR-429, OVCAR-433, IgRov and SK-OV-3, were purchased from ATCC (Manassas, VA). All cell lines were cultured in RPMI 1640 supplemented in 10% FBS under 5% CO_2_.

### Antibodies and reagents

Anti-integrin α2 (ΙΙΕ10), α3 (X8), α6 (ELE) and β1 (P5D2), along with anti-tetraspanin CD151 (5C11), CD9 (C9BB) and CD82 (M104), were raised in-house as described in prior studies [19]. The CD151-specific monoclonal antibody used for IHC analyses was obtained from Leica Microsystems, Inc. (Buffalo Grove, IL). Antibodies against fibronectin and Mucin 5Ac were purchased from Sigma-Aldrich (St. Louis, MO). Antibodies recognizing total and phospho-Akt or MAPK or GSK3-β, along with those against Snail, Slug, LRP5, LPR6 and Axin-2, were obtained from Cell Signaling Technology (Danvers, MA). Antibodies against E-cadherin, Twist, Zeb1 and Zeb2 were purchased from Santa Cruz Biotechnology (Santa Cruz, CA). Matrigel invasion chambers and anti-β4 integrin antibody were purchased from BD Biosciences (Franklin Lake, NJ). ICG-001, an inhibitor of canonical Wnt signaling, was obtained from Selleckchem (Houston, TX).

### Assays for cell proliferation, apoptosis, adhesion, motility and invasion

Changes in ovarian tumor cell proliferation or viability when subjected to Wnt inhibitor treatment were determined by MTT assay. Change in cell cycle and apoptosis was measured by staining fixed cells with propidium iodide, followed by analyses on flow cytometry. The assays for cell adhesion and invasion were carried out as previously described [19]. For measurement of random cell motility, cells were seeded into 24 well plates at 1.5 x10^4^ per well and placed inside OKALAB incubator with a heater and gas mixer constant at 37 ^0^C and 5% CO_2_. The cells were imaged at 20 min intervals for the indicated length of time with a Nikon Automated Eclipse Ti-E inverted microscope. Quantitation of individual cell movement indicated by distance, was measured by using Nikon NIS-Elements Advanced Analysis Software as previously described [18].

### RNAi, transfection, FACS, immunofluorescence analysis (IF) and IHC analyses

Stable knockdown of CD151 and Slug was carried out as previously described. The siRNA for α3 integrin was obtained from Thermo Scientific (Pittsburgh, PA) as described in our prior study and siRNA for E-Cadherin was purchased from Santa Cruz Biotechnology (Santa Cruz, CA). The shRNA for Slug was purchased from Addgene (Cambridge, MA). The surface expression of integrins and tetraspanins was determined by FACS analyses of 10,000 cells on flow cytometry at UK core facility. For indirect immunofluorescence analyses of cell surface proteins, cells were incubated with primary and fluorescence-conjugated antibodies on ice before being fixed in 2% paraformaldehyde, and visualized under a confocal microscope.

IHC analyses of TMAs and human fallopian tube tissues were conducted essentially as described in our prior study [18, 19]. In brief, the antigen was retrieved by use of the decloaking chamber in the presence of 0.01 mol/L EDTA buffer (pH 9.0). After blocking in goat serum, TMA slides were incubated with CD151-specific monoclonal antibody, followed by incubation with biotinylated secondary antibody, and finally developed using DAB Kit (BD Bioscience, San Jose, CA) and counterstained with hematoxylin. The scoring of immunostaining was evaluated on the bases of staining intensity and percentages of positively stained areas by three independent staff members as previously described [19].

### Signaling, [^3^H]-palmitate labeling, co-immunoprecipitation and western blot analyses

For evaluation of the effect of CD151 on cell signaling, semi-confluent ovarian cancer cells grown under various conditions were lysed in RIPA buffer supplemented with protease and phosphatase inhibitors, and subsequently blotted with indicated antibodies. The compositions of RIPA buffer were described elsewhere [26]. For co-immunoprecipitation of tetraspanin-integrin complexes, cultured human ovarian cancer cells were radio-labeled with 0.2 μCi [^3^H]-palmitate for 3 hours prior to being lysed in 1% Brij-96 buffer as previously described [65]. The biotin-labeling of cell surface molecules were carried out as previously described [65]. The cell lysates were then immunoprecipitated with indicated tetraspanin or integrin-specific antibodies. The immune complexes or cell lysates were separated on SDS-PAGE, transferred onto a nitrocellulose membrane and exposed to radiography or blotted with indicated antibodies.

### Xenograft analyses

Analyses of ovarian tumor growth were conducted by subcutaneously injecting control and CD151-ablated OVCAR-5 cells into the flanks of immune-compromised nude mice (Charles River, MA) at 1× 10^6^ cells per site. For analyses of ascites production, 1× 10^6^ (in 200 μl) OVCAR-5 cells were injected into the peritoneal body cavity of nude mice at 5 animals per group. During the animal studies, mice were monitored daily for the initial appearance of palpable tumors or an enlarged abdomen due to ascites production. The tumor sizes were measured twice a week with calipers and volumes were calculated using the formula of length × width × height × 0.52. All animals were terminated with the apparent appearance of disease. At the end of the experiments, tumors derived from subcutaneously injected mice were collected and fixed in 10% neutral formalin (Sigma-Aldrich, St. Louis, MO) and stored in 70% ethanol prior to being embedded into paraffin blocs for H & E staining.

### Gene expression profiling analyses

To conduct DNA array analyses, total RNA was extracted from semi-confluent human OVCAR-5 cells expressing control and CD151-specific shRNA by use of the Trizol method (Life Technologies, Grand Island, NY) and subsequently analyzed with Affiymatrix U133 PLUS 2 arrays.

### Statistical analyses

All experiments described in the study were independently repeated. The Fisher exact test was performed on the association of CD151 expression in metastatic lesions of human ovarian tumors in TMA. The paired student t-test was conducted to assess the difference in CD151 staining between 7 paired human primary ovarian tumors and metastatic lesions or the impact of CD151 knockdown on ovarian tumor growth. Difference in tumor-free survival was assessed by the log-rank test.
